# Supplementary material for: Two Gold Kiwifruit Daily for Effective Treatment of Constipation in Adults—A Randomized Clinical Trial
Source: Nutrients. 2022 Oct 6;14(19):4146. doi: 10.3390/nu14194146 (PMC9572406; doi:10.3390/nu14194146)
Supplement: Supplementary file 1 [file nutrients-14-04146-s001.zip › Suppemental Table S2.pdf]

Supplemental Table S2 Adverse events. Total counts, details, and follow up information[a]

Phase (n) [statistical significance][b]	Serious/ Non-serious (n)	Possibly intervention related [statistical significance]	Details (n) [statistical significance]	Withdrawals	Recovered
Psyllium Intervention (22)<br>[*P*=0.0468]	non-serious (22)	18<br>[P=0.0155]	Cramping (3)<br>Constipation (3)<br>Struggle to swallow (3)<br>Rhinovirus (2)<br>Excessive thirst (1)<br>Bloating (5)<br>Abdominal discomfort (2)<br>Viral gastroenteritis (1)<br>Belching (1)<br>Migraine (1)<br>Erratic bowel motions (1)<br>Diarrhea (1)	1	19<br>Unknown 3[c]
Kiwifruit Intervention (11)<br>[*P*=0.0468]	non-serious (10)<br>serious (1)	7<br>[P=0.0155]	Allergic Rash (1)<br>Rhinovirus (1)<br>Post-Surgical Bacterial Infection of the Knee (1)<br>Viral Gastroenteritis (1)<br>Bloating (2)<br>Diarrhea (2)<br>Constipation (1)<br>Heart burn (1)<br>Anxiety due to probiotic withdrawal (1)	2	11
Wash-out (6)<br>Lead in (6)<br>Follow up (3)	non-serious (10)	2	Rhinovirus (3)<br>Bacterial Infections and Antibiotics (1)<br>Viral Gastroenteritis (1)<br>Aspirin Gastritis (1)<br>Urine tract infection (1)<br>Internal Hemorrhoid (1)<br>Passing a blood clot (1)<br>Knee sprain (1)	0	10

[a] Values are presented as total counts. Multiple adverse events per person recorded.
[b] Unpaired t-test, n=73
[c] Participant ignored attempts for follow up and was ultimately withdrawn.
