# Supplementary material for: Two Gold Kiwifruit Daily for Effective Treatment of Constipation in Adults—A Randomized Clinical Trial
Source: Nutrients. 2022 Oct 6;14(19):4146. doi: 10.3390/nu14194146 (PMC9572406; doi:10.3390/nu14194146)
Supplement: Supplementary file 1 [file nutrients-14-04146-s001.zip › Supplemental Table S1.pdf]

Supplemental Table S1 Impact of Interventions on PROMIS and SAGIS scores post – pre-intervention

Domain	Group	SunGold® Kiwifruit	BonVit® Psyllium	*P* value Factor (*n* = 54)		
				Effect of Interventions[a)]	Gold kiwifruit vs psyllium[b)]	Participant group effects[c)]
PROMIS Constipation	Controls	-0.86 (1.46, -3.18)	0.08 (2.42, -2.26)			
	FC	-7.04 (-3.08, -10.99)	-1.72 (2.28, -5.72)	***F*[1,51]=6.246, *P*=0.016***	*F*[1,51]=0.484, *P*=0.490	*F*[2,51]=2.238, *P*=0.117
	IBS-C	1.12 (5.07, -2.84)	-2.10 (1.90, -6.10			
PROMIS Diarrhea	Controls	2.17 (4.79, -0.46)	0.60 (2.72, -1.51)			
	FC	0.95 (5.42, -3.53)	1.97 (5.58, -1.63)	***F*[1,51]=7.834, *P*=0.007****	*F*[1,51]=0.628, *P*=0.432	*F*[2,51]=0.526, *P*=0.594
	IBS-C	4.98 (9.46, 0.50)	2.23 (5.83, -1.38)			
PROMIS Disrupted Swallowing	Controls	-0.02 (1.76, -1.80)	0.39 (1.57, -0.78)			
	FC	-1.26 (1.77, -4.30)	-3.54 (-1.53, -5.54)	***F*[1,51]=4.653, *P*=0.036***	*F*[1,51]=0.373, *P*=0.544	*F*[2,51]=2.968, *P*=0.060
	IBS-C	-2.73 (0.31, -5.76)	0.67 (2.67, -1.33)			
PROMIS Belly Pain (*n*=48)	Controls	1.97 (5.23, -1.30)	0.67 (4.14, -2.80)			
	FC	-2.62 (2.96, -8.19)	-1.21 (4.70, -7.12)	*F*[1,51]=0.175, *P*=0.678	*F*[1,51]=0.093, *P*=0.761	*F*[2,51]=0.255, *P*=0.776
	IBS-C	0.06 (5.64, -5.51)	-1.73 (4.19, -7.64)			
PROMIS Bloating (*n*=41)	Controls	-2.33 (0.24, -4.90)	0.25 (2.62, -2.13)			
	FC	-1.09 (3.97, -6.15)	-2.55 (2.12, -7.22)	*F*[1,45]=1.318, *P*=0.257	*F*[1,45]=0.477, *P*=0.493	*F*[2,45]=0.483, *P*=0.620
	IBS-C	-1.03 (3.74, -5.80)	1.68 (6.08, -2.73)			
PROMIS Nausea	Controls	0.73 (3.34, -1.88)	1.04 (2.57, -0.48)			
	FC	-1.25 (3.21, -5.70)	1.30 (3.90, -1.30)	*F*[1,51]=0.857, *P*=0.359	*F*[1,51]=3.917, *P*=0.053	*F*[2,51]=1.195, *P*=0.311
	IBS-C	-5.06 (-0.61, -9.51)	-0.62 (1.98, -3.22)			
PROMIS Reflux	Controls	0.29 (1.88, -1.29)	0.66 (2.70, -1.38)			
	FC	-3.06 (0.06, -6.17)	-3.79 (0.22, -7.79)	*F*[1,38]=2.638, *P*=0.113	*F*[1,38]=2.295, *P*=0.138	*F*[2,38]=2.381, *P*=0.106
	IBS-C	-3.13 (-0.01, -6.24)	3.20 (7.20, -0.80)			
PROMIS Anxiety	Controls	-1.13 (1.38, -3.65)	-1.01 (1.85, -3.87)			
	FC	-1.00 (3.29, -5.29)	-1.55 (3.33, -6.42)	*F*[1,51]=3.265, *P*=0.077	*F*[1,51]=0.016, *P*=0.899	*F*[2,51]=0.056, *P*=0.945
	IBS-C	-2.28 (2.01, -6.58)	-1.18 (3.70, -6.06)			
PROMIS Depression	Controls	-0.91 (1.06, -2.87)	-1.69 (0.45, -3.83)			
	FC	-0.68 (2.67, -4.03)	-1.32 (2.33, -4.97)	*F*[1,51]=2.009, *P*=0.162	*F*[1,51]=0.065, *P*=0.799	*F*[2,51]=0.051, *P*=0.951
	IBS-C	0.09 (3.44, -3.26)	0.37 (4.02, -3.28)			
SAGIS Epigastric Symptoms	Controls	-0.01 (0.11, -0.13)	0.07 (0.20, -0.06)			
	FC	-0.21 (0.00, -0.41)	-0.22 (0.00, -0.44)	***F*[1,51]=10.001, *P*=0.003****	*F*[1,51]=0.015, *P*=0.903	*F*[2,51]=0.292, *P*=0.748
	IBS-C	-0.14 (0.06, -0.35)	-0.18 (0.04, -0.40)			
SAGIS Acid regurgitation /Gas	Controls	-0.02 (0.08, -0.12)	0.03 (0.11, -0.05)			
	FC	-0.09 (0.08, -0.26)	-0.15 (-0.02, -0.29)	***F*[1,51]=11.478, *P*=0.001*****	*F*[1,51]=0.996, *P*=0.323	*F*[2,51]=1.144, *P*=0.327
	IBS-C	-0.24 (-0.07, -0.41)	-0.06 (0.07, -0.20)			
SAGIS IBS/ Diarrhea	Controls	0.08 (0.20, -0.04)	0.02 (0.13, -0.10)			
	FC	-0.14 (0.07, -0.34)	0.08 (0.27, -0.12)	*F*[1,51]=0.000, *P*=0.982	*F*[1,51]=0.018, *P*=0.892	*F*[2,51]=1.794, *P*=0.177
	IBS-C	0.08 (0.28, -0.13)	-0.11 (0.09, -0.30)			
SAGIS Nausea/ Vomiting	Controls	0.08 (0.27, -0.11)	0.06 (0.17, -0.05)			
	FC	-0.16 (0.16, -0.48)	-0.16 (0.03, -0.35)	F[1,51]=0.703, p=0.406	F[1,51]=0.004, p=0.948	F[2,51]=0.006, p=0.994
	IBS-C	-0.05 (0.27, -0.36)	-0.05 (0.14, -0.24)			
SAGIS Constipation	Controls	-0.02 (0.20, -0.23)	0.09 (0.31, -0.12)			
	FC	-0.68 (-0.32, -1.05)	-0.41 (-0.05, -0.77)	***F*[1,51]=11.157, *P*=0.002****	*F*[1,51]=0.234, *P*=0.631	***F*[2,51]=4.178, *P*=0.021***
	IBS-C	0.05 (0.41, -0.32)	-0.50 (-0.14, -0.86)			

Data are presented as Δ-score = median (upper bound, lower bound 95% intervals) after subtraction of pre-intervention score from post-intervention score = relative shift from baseline. Δ-Scores were compared by two-way ANOVA. Bold data represent statistical significance. *P<0.05, **P<0.01, ***P≤ 0.001. FC, Functional Constipation, IBS-C, constipation-predominant Irritable Bowel Syndrome, SAGIS, Structured Assessment of Gastrointestinal Symptoms, PROMIS, Patient reported Outcome Measurement Information System. [a)] Combined effect of interventions on the relative shift from baseline; [b)]Kiwifruit vs psyllium = differences between the interventions on their effect on the relative shift; [c)]Participant group effects on the relative shift from baseline.
